# Supplementary material for: SGLT2 inhibitors attenuate endothelial to mesenchymal transition and cardiac fibroblast activation
Source: Sci Rep. 2024 Jul 16;14:16459. doi: 10.1038/s41598-024-65410-9 (PMC11252266; doi:10.1038/s41598-024-65410-9)
Supplement: Supplementary file 2 — Supplementary Information 2. [file 41598_2024_65410_MOESM2_ESM.pdf]

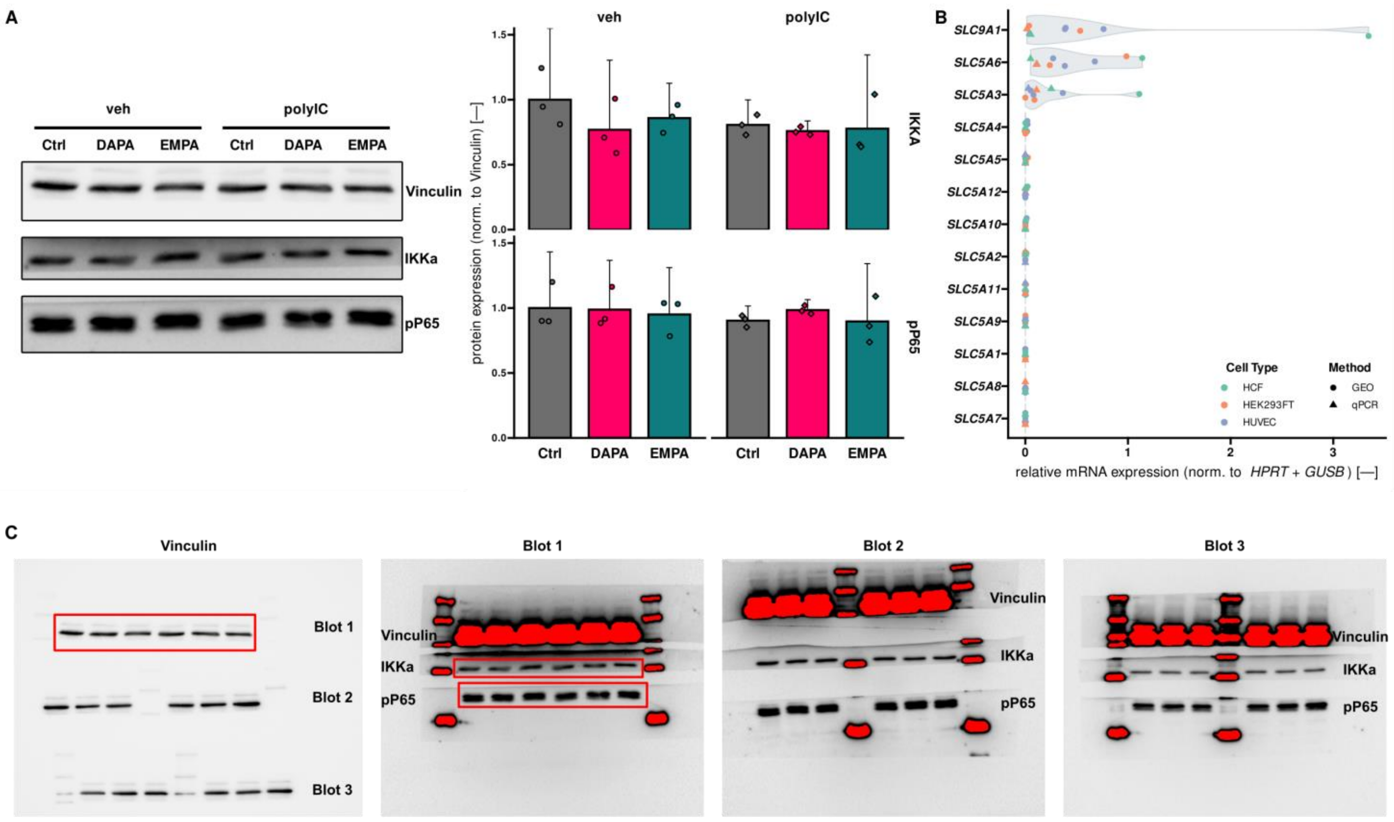

Supplemental Figure S1: (A) Representative western blot of inhibitor of kappa-B kinase α (IKKA) and phosphorylated P65 (pP65) in human umbilical vein endothelial cellls (HUVECs) treated with 100 µM DAPA or EMPA or the respective control (Ctrl) (left) and quantification of band intensity normalized (norm.) to vinculin (right, $n = 3$). **(B)** Expression levels of solute carrier (*SLC*) 5A family and *SLC9A1* in cell types used in this study. Shapes of individual points indicate the utilized method. **(C)** Uncropped images of Western Blots. Red rectangles highlight bands shown exemplarily in **(A)**. veh, vehicle; polyIC, polyinosinic:polycytidylic acid; norm., normalized; HCF, human cardiac fibroblast; HEK, human embryonal kidney; GEO, gene expression omnibus; qPCR, quantitative real-time polymerase chain reaction; *HPRT*, hypoxanthine-guanine phosphoribosyltransferase; *GUSB*, β-glucuronidase.

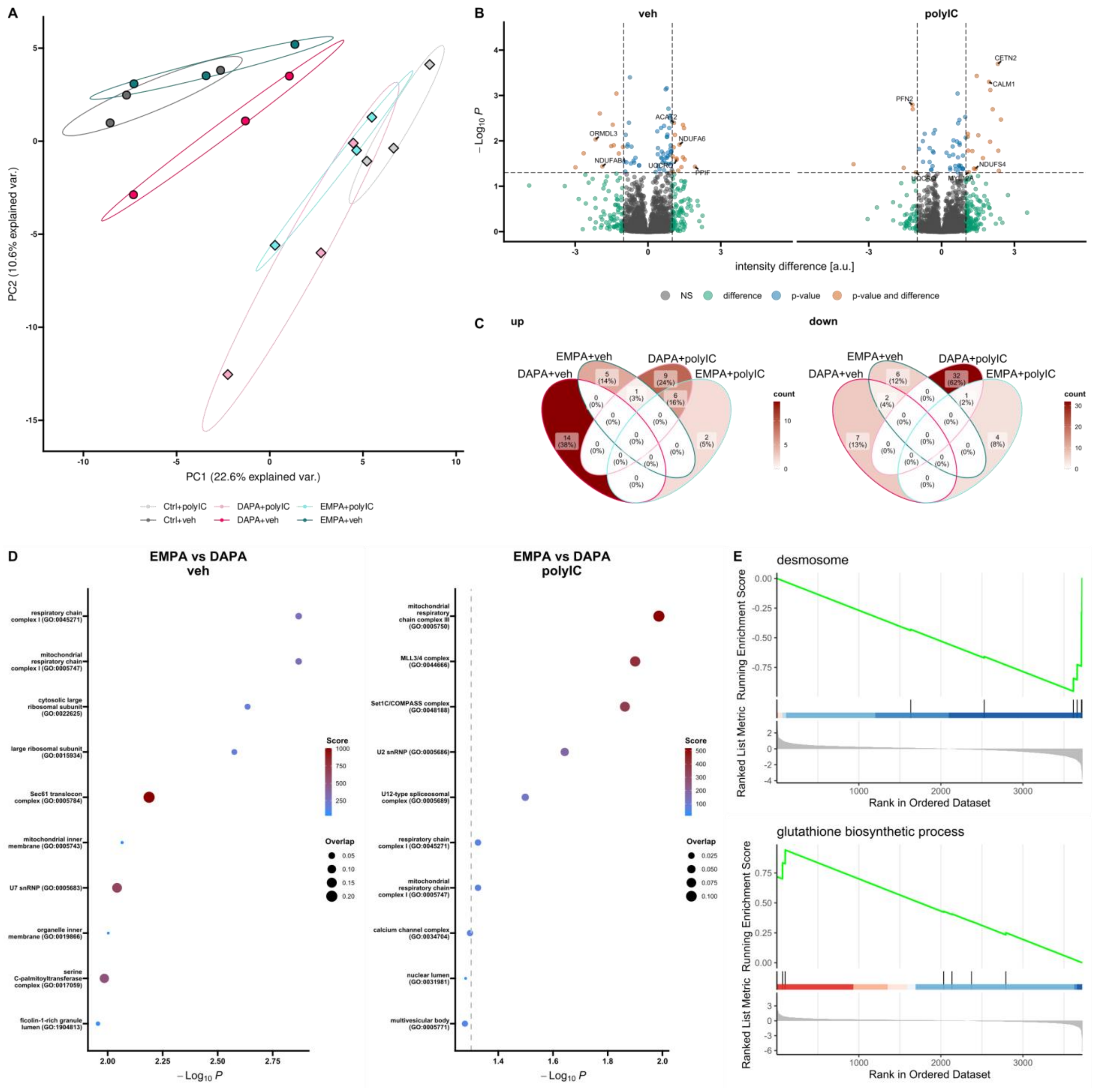

Supplemental Figure S2: (A) Principal component (PC) analysis of proteomics data generated from human umbilical vein endothelial cells (HUVECs) treated with dapagliflozin (DAPA) or empagliflozin (EMPA) or control (Ctrl). Individual biological replicates are represented as dots, ellipses group by treatment (*n* = 3). **(C)** Volcano plots depicting dysregulated proteins in EMPA versus DAPA comparisons in vehicle (veh) and polyinosinic:polycytidylic acid (polyIC) exposed HUVECs. NS, non-significant; a.u., arbitrary units. **(C)** Venn-diagrams highlighting the overlap of up- (left) and downregulated (right) proteins compared to the respective Ctrl of different groups in proteomics experiments. **(D)** Analysis of dysregulated proteins under comparison of EMPA and DAPA in veh (left) and polyIC (right) stimulated HUVECs for overrepresentation of gene ontology (GO) terms. Analysis was done with Enrichr. Shown are top ten overrepresented terms by p-value. Dashed lines indicate p-value cutoff. **(E)** Gene set enrichment analysis of selected

terms for DAPA (top) and EMPA (bottom) versus Ctrl in polyIC treated HUVECs. Relevant terms are plotted, ranking was based on respective expression differences.

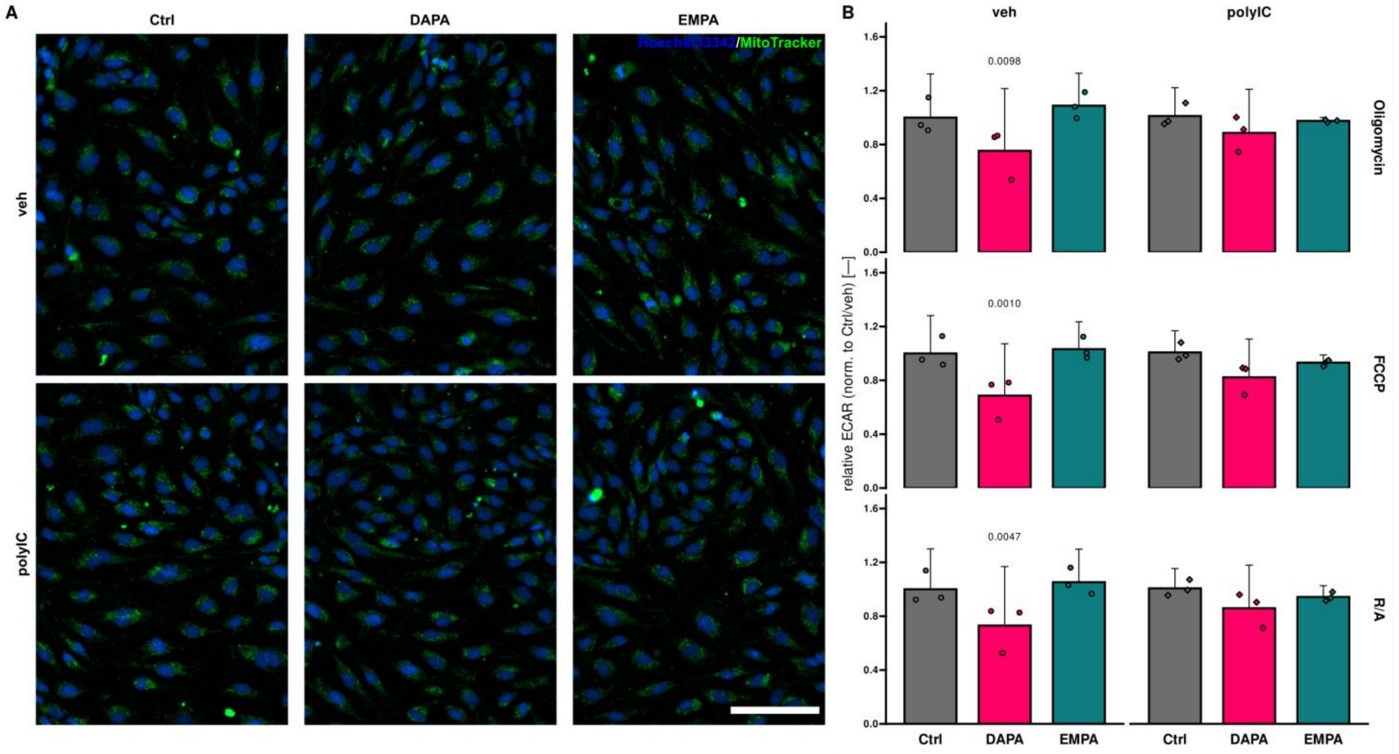

Supplemental Figure S3: (A) Representative images of HUVECs receiving the treatment as indicated. Mitochondria are labelled with MitoTracker Green dye and Nuclei are stained with Hoechst33342 (n = 3). Scale bar, 100 µM. **(B)** Extracellular acidification rate (ECAR) values of HUVECs normalized (norm.) to Control (Ctrl) / vehicle (veh) group at the different stages of Seahorse MitoStress test assay (n = 3). polyIC, polyinosinic:polycytidylic acid; FCCP, carbonyl cyanide-p-trifluoromethoxyphenylhydrazone; R/A, rotenone/antimycin A, DAPA, dapagliflozin; EMPA, empagliflozin.

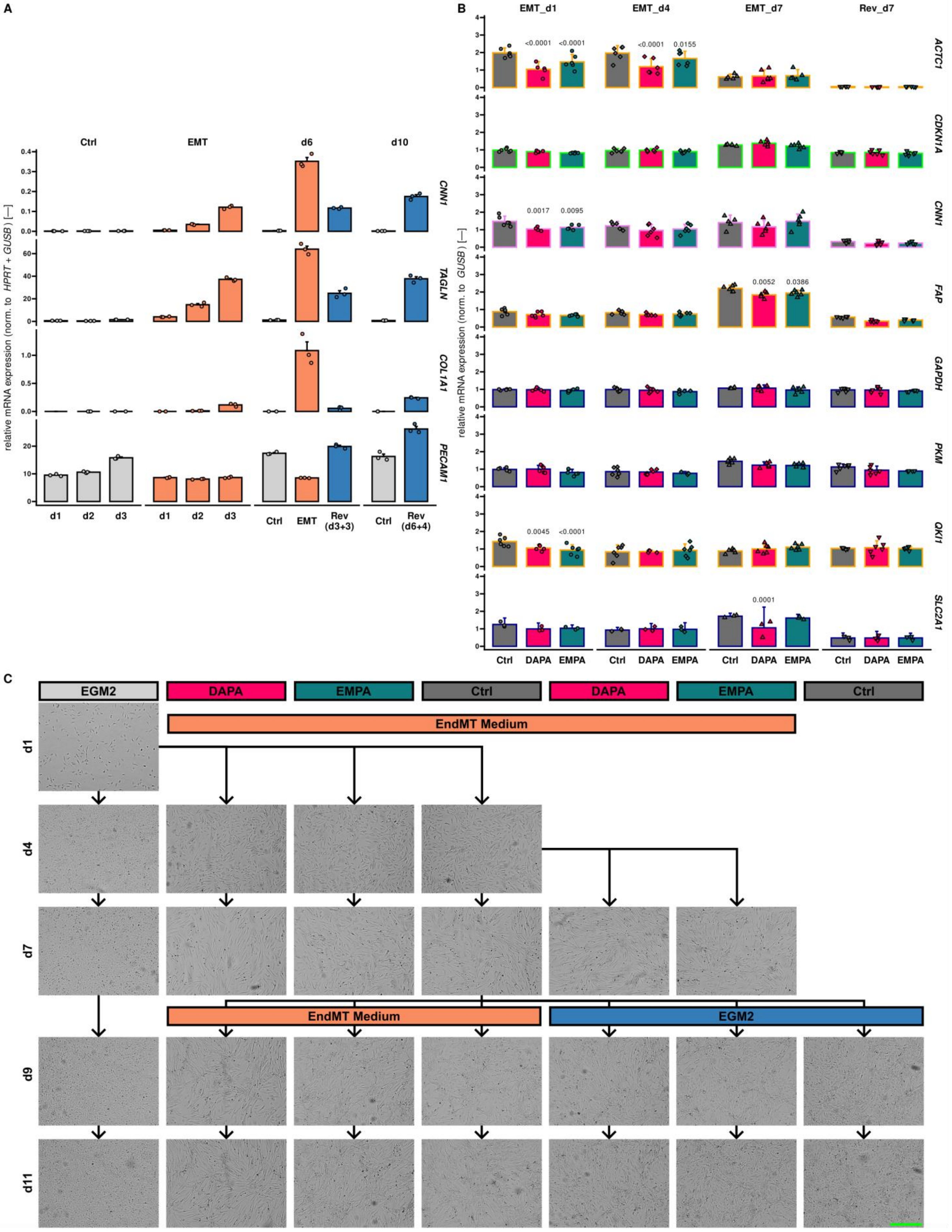

Supplemental Figure S4: (A) Relative expression of genes indicating endothelial to mesenchymal transition (EndMT). Human umbilical vein endothelial cells (HUVECs) were seeded on day (d) 0, 24 h prior to exchange of media to either endothelial cell medium (Ctrl) or EndMT medium (EMT) maintained for indicated times or changed back to Ctrl as

labelled (Rev) (3 technical replicates). **(B)** Relative expression of various genes for assessed categories in HUVECs undergoing EndMT ($n \geq 4$). **(D)** Examplary bright field images of HUVECs undergoing EndMT. Days of recordings are indicated on the left, seeding was done on d0. Scale bar (green), 500 µm (n = 3). DAPA, dapagliflozin; EMPA, empagliflozin; Ctrl, control; *HPRT*, hypoxanthine-guanine phosphoribosyltransferase; *GUSB*, β-glucuronidase; *CNN*, calponin; *TAGLN*, transgelin; *PECAM*, platelet endothelial cell adhesion molecule; *COL*, collagen; *ACTC1*, cardiac muscle alpha actin; *FAP*, fibroblast activating protein; *GAPDH*, glyceraldehyde 3-phosphate dehydrogenase; *CDKN*, cyclin-dependent kinase inhibitor; *PKM*, pyruvate kinase, muscle; *QKI*, quaking; *SLC*, solute carrier.

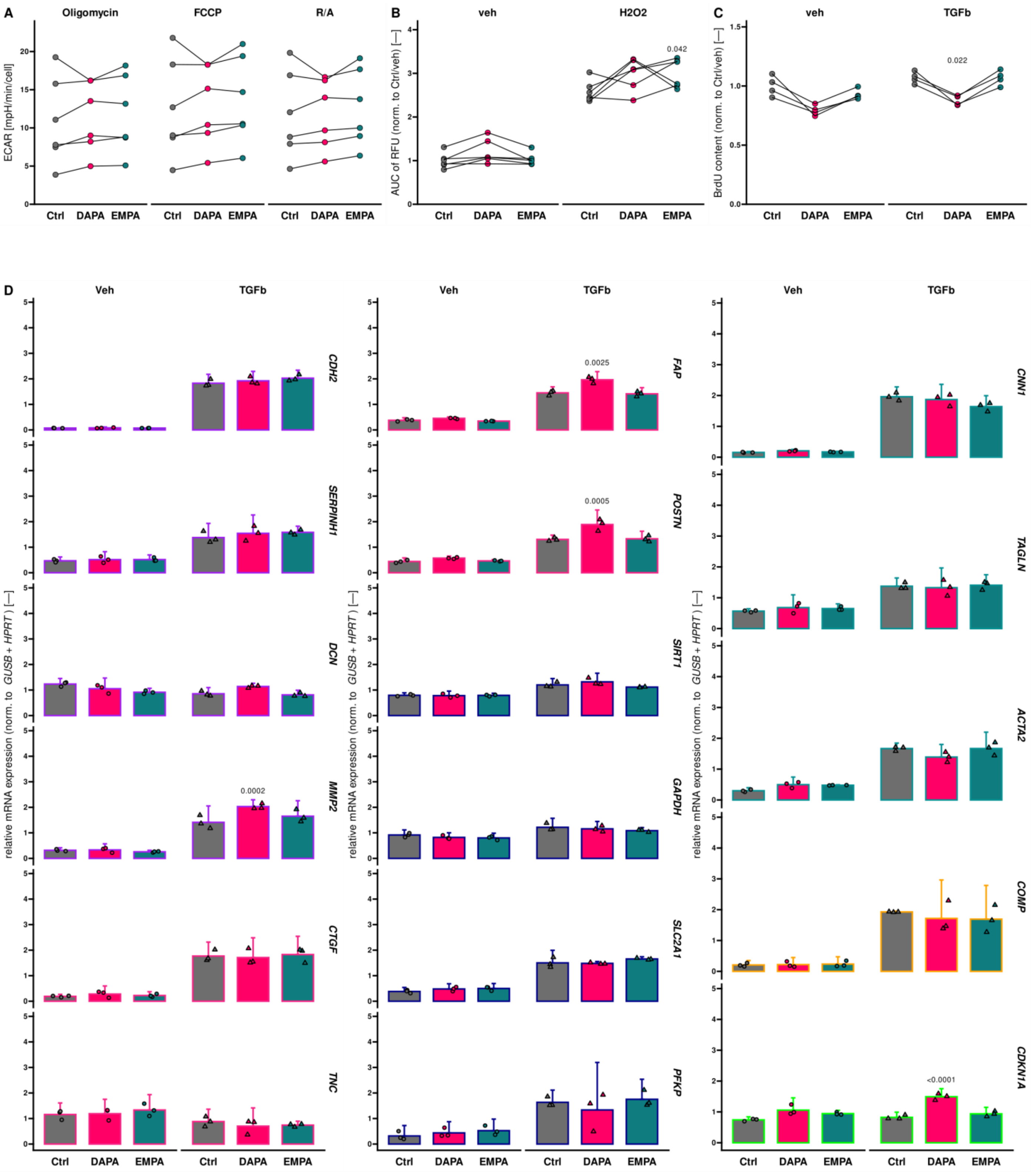

Supplemental Figure S5: **(A)** Extracellular acidification rate (ECAR) values at the different stages of Seahorse MitoStress test assay of human cardiac fibroblasts (HCFs) (*n* = 6). FCCP, carbonyl cyanide-p-trifluoromethoxyphenylhydrazone; R/A, rotenone/antimycin A. **(B)** Area under curve (AUC) of relative fluorescence units (RFU) curves depicting reactive oxygen species levels in HCFs treated as described assessed with H2DCFDA assay (*n* = 6). **(C)** Relative content of 5-bromo-2'-deoxyuridine (BrdU) incorporated over 24 h into DNA of replicating (treated) HCFs normalized to control (Ctrl) treated, vehicle (veh) receiving cells (*n* = 4). **(D)** Relative expression of

genes in HCFs for various assessed fibrosis-associated categories (*n* = 3). DAPA, dapagliflozin; EMPA, empagliflozin; Ctrl, control; TGFb, transforming growth factor β; *HPRT*, hypoxanthine-guanine phosphoribosyltransferase; *CDKN*, cyclin-dependent kinase inhibitor; *COMP*, Cartilage oligomeric matrix protein; *PFKP*, Phosphofructokinase, platelet; *POSTN*, periostin; *GUSB*, β-glucuronidase; *CNN*, calponin; *TAGLN*, transgelin; *ACTA2*, α smooth muscle actin; *FAP*, fibroblast activating protein; *GAPDH*, glyceraldehyde 3-phosphate dehydrogenase; *CDKN*, cyclin-dependent kinase inhibitor; *SLC*, solute carrier; *CDH*, cadherin; *DCN*, decorin; *MMP*, matrix metalloproteinase; *CTGF*, connective tissue growth factor; *TN*, tenascin; *SIRT*, sirtuin.

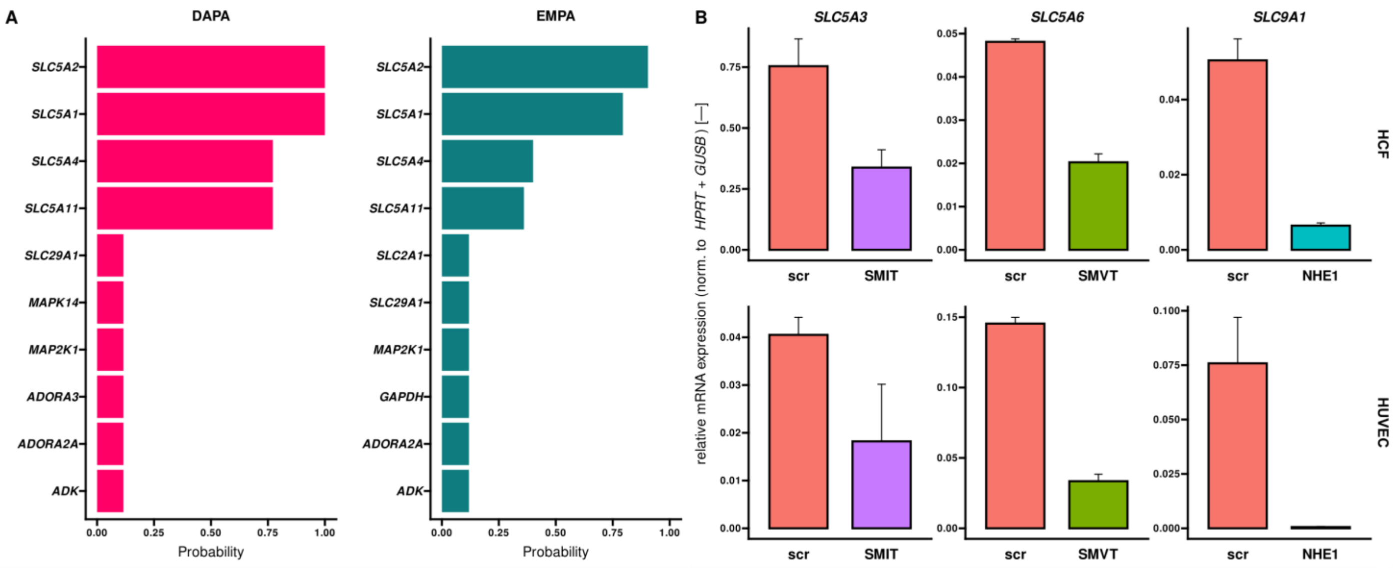

Supplemental Figure S6: (A) Probability of interaction of dapagliflozin (DAPA) and empagliflozin (EMPA) with respective targets according to SwissTargetPrediction webtool. Top ten candidates are listed. **(B)** SiRNA mediated knockdown of expression of respective genes in human cardiac fibroblasts (HCF) and human umbilical vein endothelial cells (HUVEC) on mRNA level assessed with quantitative real time polymerase chain reaction (3 technical replicates). *SLC*, solute carrier; *GUSB*, β-glucuronidase; *HPRT*, hypoxanthine-guanine phosphoribosyltransferase; SMIT, sodium-myoinositol cotransporter ; SMVT, sodium-multivitamin cotransporter; NHE, sodium proton exchanger.

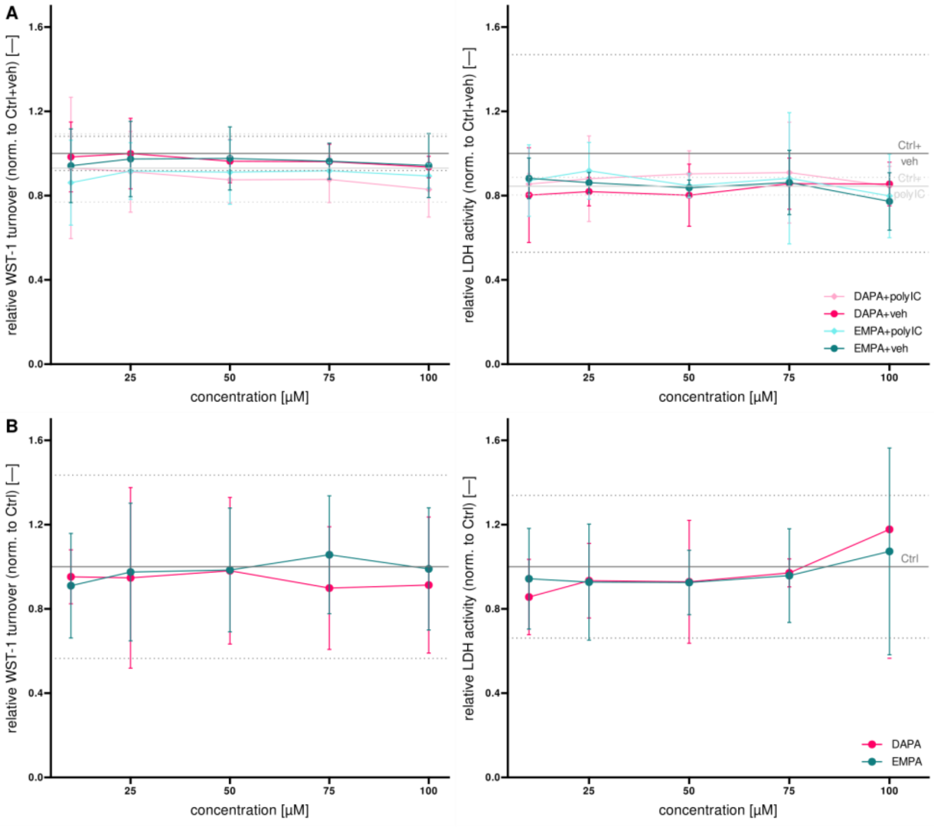

Supplemental Figure S7: (A, B) Viability assessment of human umbilical vein endothelial cells **(A)** and human cardiac fibroblasts **(B)** done with measurement of water soluble tetrazolium (WST)-1 turnover (left) within cells and lactate dehydrogenase (LDH) activity in supernatants of cultured cells treated as indicated (*n* = 3). Norm., normalized; polyIC, polyinosinic:polycytidylic acid; veh, vehicle; Ctrl, control; DAPA, dapagliflozin; EMPA, empagliflozin.

Supplemental Material and Methods

Western Blot

HUVECs were seeded at a density of 150000 cells per well in 6-well plates (TPP) and treated for 48 h in EGM-2. Cells were trypsinized, two wells were pooled, and pelleted as described. After washing once with PBS, HUVECs were lysed in 1× Cell Lysis Buffer (#9803, Cell Signaling Technology, Danvers, MA, USA) containing 200 mg/mL Pefabloc® SC (76307, Sigma-Aldrich) for 15 min on ice. Cellular remnants were removed by centrifugation (8000 ×g, 5 min, 4 °C) and protein concentration was determined with Bradford assay by diluting the sample in 1× Roti-Quant (K015, Carl Roth) and measuring the optical density at 595 nm at the Synergy HT. For quantification, a standard curve using BSA Fraction V (8076, Carl Roth) was created. Sodium dodecyl sulfate polyacrylamide gel electrophoresis (SDS-PAGE) gel was cast by mixing ddH_2O, 1.5 M Tris (pH 8.8) containing 0.4% (w/v) SDS, Rotiphorese Gel 30 (Carl Roth), ammonium persulfate (10% w/v in H_2O), and Temed (Carl Roth) in a volumetric ratio of 700:390:400:10:1. Collection gel was prepared by combining ddH_2O, 1 M Tris (pH 6.8) containing 0.4% (w/v) SDS, Rotiphorese Gel 30, 10% (w/v) ammonium persulfate, and Temed in a volumetric ratio of 3800:1600:820:50:3. Per sample, 20 µg of protein was prepared for gel loading by mixing with an appropriate volume of loading buffer containing dithiothreitol (DTT, both New England Biolabs, Ipswich, MA, USA), then heated to 95 °C for 5 min for denaturation and subsequently cooled on ice. Samples and Precision Plus Protein WesternC Standards (Bio-Rad) were loaded into gel wells and SDS-PAGE was run at 30 mA for approximately 1.5 h. Afterwards, separated proteins were blotted onto a polyvinylidene fluoride (Bio-Rad) membrane over night at 4 °C and 30 V. The membrane was then blocked with 5% (w/v) milk powder in Tris-buffered saline (20 mM, pH 7.5) containing 0.1% (v/v) Tween-20 (TBST, all Carl Roth) for 1 h at RT and incubated in respective primary antibody solutions (see **Supplemental Table S1**) at 4 °C over night. After washing the membrane thrice in TBST for 5 min at RT, incubation in secondary antibody solution (see **Supplemental Table S1**) was performed for 1 h at RT. The membrane was washed three times in TBST for 5 min at RT, and Clarity Western ECL substrate (Bio-Rad) was used according to manufacturer's instructions to visualize bands. Chemiluminescent signals were recorded with a ChemiDoc device (Bio-Rad) and analyzed using Fiji [1].

Cellular viability

Cells were seeded into 96-well plates at a concentration of 10000 cells per well. After 24 h treatments were applied as outlined before at a volume of 100 µL per well. At the end of the treatment duration, a positive control for cellular decay was generated by addition of 1:100 Lysis Solution (from CellTox™ Green Cytotoxicity Assay, Promega) for 10 min at

37 °C. Afterwards, 50 µL of the cell culture supernatant was removed after gentle mixing by pipetting, avoiding detachment of viable cells. The removed volume was processed according to the CytoTox 96® Non-Radioactive Cytotoxicity Assay (Promega) protocol measuring lactate dehydrogenase (LDH) activity. Absorbance was read at a Synergy HT at 490 nm and values were normalized to the respective lysis control prior to further processing. Remaining medium was removed from cells and water soluble tetrazolium (WST)-1 Cell Proliferation Reagent (Roche) diluted 1:10 in respective culture medium was added. Optical density at 450 nm was measured after 1 h to 2 h using a Cytation1 / BioSpa8 combination and background (at 630 nm) was subtracted from values before further analysis.

MitoTracker Staining

HUVECs were seeded into a 96-well plate at a density of 10000 cells/well and treated as described. After treatment end, cells were stained with 500 nM MitoTracker Green (Thermo Fisher Scientific) and 5 µg/mL Hoechst33342 in OptiMEM for 15 min at 37 °C. Images were captured using a Cytation1 in "DAPI" ($\lambda_{\mathrm{Excitation}} = 377$ nm, $\lambda_{\mathrm{Emission}} = 447$ nm) channel and "GFP" channel ($\lambda_{\mathrm{Excitation}} = 469$ nm, $\lambda_{\mathrm{Emission}} = 525$ nm).

References

1. Schindelin, J. *et al.* Fiji: an open-source platform for biological-image analysis. *Nat. Methods* **9**, 676 (2012).

Supplemental Table S1: Antibodies used in this project.

Target	Species	Manufacturer	Concentration
Vinculin	Mouse	Sigma Aldrich, V9131	1:30000
IKKA	Rabbit	Cell Signaling, 2682	1:1000
pP65	Rabbit	Cell Signaling, 3033	1:1000
Mouse IgG	Donkey	Cell Signaling, 7076	1:10000
Rabbit IgG	Donkey	Cell Signaling, 7074	1:10000

IKKA, inhibitor of kappa-B kinase α; pP65, phosphorylated P65; IgG, immunoglobulin G.

Supplemental Table S2: Sequences of primer pairs used in this project.

Target	Forward sequence (5' to 3')	Reverse sequence (5' to 3')
ACTA2	CCTGACTGAGCGTGGCTATT	GATGAAGGATGGCTGGAACA
ACTC1	AGCCCTCCTTCATTGGTATGG	CGCTCAGGGGGAGCAATAAT
CDH2	AAGAGACCCAGGAAAAGTGGC	TCTGCTGACTCCTTCACTGACT
CDKN1A	GCAGACCAGCATGACAGATTTC	GGATTAGGGCTTCCTCTTGGA
CDKN2A	Qiagen QuantiTect QT00089964	
CNN1	GCCCCACGACATTTTTGAGG	CTTTCGTCTTCGCCATGCT
COL1A1	ACGAAGACATCCCACCAATC	CTTGGTCGGTGGGTGACTCT
COMP	CCCAGAAGAACGACGACCAA	GTCCACATCCGCCTGATCC
CTGF	GTGTGCACCGCCAAAGAT	GTGTCTTCCAGTCGGTAAGC
DCN	GCATAAGTACATCCAGGTTGTCT	CCGGGTTGCTGAAAAGACTC
FAP	TCCTGGCTTCAGCTTCCAAC	AGGGCGTAAGACAATGCACA
GAPDH	CCAGGCGCCCAATACG	CCACATCGCTCAGACACCAT
GUSB	GACACCCACCACCTACATCG	CTTAAGTTGGCCCTGGGTCC
HPRT	AGGACTGAACGTCTTGCTCG	GTCCCCTGTTGACTGGTCATT
ICAM1	GGCTGGAGCTGTTTGAGAAC	ACTGTGGGGTTCAACCTCTG
IL6	ACCCCCAGGAGAAGATTCCA	TGCCTCTTTGCTGCTTTCAC
MMP2	TGACATCAAGGGCATTCAGGAGC	GTCCGCCAAATGAACCGGTCCTTG
PECAM1	CCTTCTGCTGAGCGAGTCAT	CATTTTGCACCGTCCAGTCC

PFKP	AGGAGCTGGCCAGGAACG	CGTCCCATCACCTCCAGAAC
PKM	TCCGGATCTCTTCGTCTTTGC	GCTGGGTCTGAATGAAGGCA
POSTN	TAGTCGTATCAGGGGTCGGG	TGGGCAGCCTTTCATTCCTT
QKI1	CAGCCCTTGCCTTTTCTCTTGC	ATAGGTTAGTTGCCGGTGGC
SERPINH1	ATCAACGAGTGGGCCGC	ATCCCAGTGTGGCTTGAAGAA
SIRT1	TCGCAACTATACCCAGAACATAGACA	CTGTTGCAAAGGAACCATGACA
SLC2A1	TTGGCTCCGGTATCGTCAAC	GATGGCCACGATGCTCAGAT
SLC5A1	TCCGCAAGAGAGCATCTGAG	AGGCGATGCTGATGCCAATC
SLC5A2	TCTCTTCGCCAGCAACATCG	CACGAAGAGCGCATTCCAC
SLC5A3	AGCACCGTGAGTGGATACTTC	CCCTGACCGGATGTAAATTGG
SLC5A4	AGAGTCAATGAACAGGGAGCC	TTGCCAGGACGACCAGTATG
SLC5A5	TCCAAGGGGCTCTCACTCATC	CGCTGATGACTCCCATGACG
SLC5A6	GACTACCTTCTCCAAGCCCACAG	TGGTGGAGTTGTGAGCACTG
SLC5A7	GTTCTGGGCTGCAGCAATTT	GTGACAATGCAAAGGGGACG
SLC5A8	TGTATTGGAATGGCTGCGCT	CATAAGCGGTCCACCAACCA
SLC5A9	CATCAGCGTGGTGGTCATCT	TACACAGGGACGAAGACCCA
SLC5A10	TGTCCTGTCCCTGCTACTGT	CAGGGCGTCCGTGTAGATTA
SLC5A11	TCCCCATCATCCTGGCTGTA	TCCGTGTAGATCACAGCAGC
SLC5A12	CTTGCTGGGTCTCTGGATCA	AAGTACGGCATCAGCTGGTCT
SLC9A1	CAACTGCCAGCACCATTCG	CCGGGACGATGCTTGAGATA
TAGLN	GTACCCTGATGGCTCCAAGC	ATGCTCCTGCGCTTTCTTCATA
TNC	GCCCATTACAGGAGGTACACC	TCAAAGCCCTTCATGGCGAT

ACTA, alpha smooth muscle actin; *ACTC*, cardiac muscle alpha actin; *CDH*, cadherin; *CDKN*, cyclin-dependent kinase inhibitor; *CNN*, calponin; *COL*, collagen; *COMP*, cartilage oligomeric matrix protein; *CTGF*, connective tissue growth factor; *DCN*, decorin; *FAP*, fibroblast activation protein alpha; *GAPDH*, glyceraldehyde-3-phosphate dehydrogenase; *GUSB*, β-glucuronidase; *HPRT*, hypoxanthine-guanine phosphoribosyltransferase; *ICAM*, intercellular adhesion molecule; *IL*, interleukin; *MMP*, matrix metalloprotease; *PECAM*, platelet endothelial cell adhesion molecule; *PFKP*, phosphofructokinase, platelet; *PKM*, pyruvate kinase M; *POSTN*, periostin; *QKI*, KH domain containing RNA binding protein QKI; *SERPINH1*, serpin family H member 1; *SIRT*, sirtuin; *SLC*, solute carrier; *TAGLN*, transgelin; *TNC*, tenascin C.
